# Outcomes of Nivolumab Plus Ipilimumab After Atezolizumab Plus Bevacizumab in Advanced HCC: An International Multicentre Study

**DOI:** 10.1111/liv.70493

**Published:** 2025-12-26

**Authors:** Jung Sun Kim, Jeffrey Wong, San‐Chi Chen, David Tai, Hannah Yang, Youngun Kim, Beodeul Kang, Ilhwan Kim, Hyeyeong Kim, Chansik An, Su Jin Jang, Masatoshi Kudo, Ho Yeong Lim, Chan Kim, Thomas Yau, Hong Jae Chon

**Affiliations:** ^1^ Medical Oncology, Department of Internal Medicine CHA Bundang Medical Center, CHA University School of Medicine Seongnam Korea; ^2^ CHA University School of Medicine Seongnam Korea; ^3^ Department of Medicine, Queen Mary Hospital The University of Hong Kong Hong Kong China; ^4^ Department of Oncology Taipei Veterans General Hospital Taipei Taiwan; ^5^ School of Medicine, College of Medicine, National Yang Ming Chiao Tung University Taipei Taiwan; ^6^ Division of Medical Oncology National Cancer Centre Singapore Singapore; ^7^ Division of Oncology, Department of Internal Medicine Inje University College of Medicine, Haeundae Paik Hospital Busan Korea; ^8^ Department of Internal Medicine Ulsan University Hospital, University of Ulsan College of Medicine Ulsan Korea; ^9^ Department of Radiology CHA Bundang Medical Center Seongnam Korea; ^10^ Department of Nuclear Medicine CHA Bundang Medical Center Seongnam Korea; ^11^ Department of Gastroenterology and Hepatology Kindai University Faculty of Medicine Osaka Japan

**Keywords:** hepatocellular carcinoma, immunotherapy, ipilimumab, nivolumab

## Abstract

**Background/Aims:**

Immune checkpoint inhibitors (ICIs) have transformed advanced HCC treatment. The benefit of sequential immunotherapy after prior ICI failure remains unclear. Given the expanded use of atezolizumab plus bevacizumab (Ate/Bev) over the past 5 years, we explored the real‐world outcomes of nivolumab plus ipilimumab (Nivo/Ipi) in patients with advanced HCC, with more focus on those previously exposed to Ate/Bev.

**Methods:**

Patients treated with Nivo/Ipi for advanced HCC from six referral hospitals in Korea, Hong Kong, Taiwan and Singapore were included. Patients with prior non–Ate/Bev ICI or Child–Pugh B–C were excluded. Outcomes were compared between the ICI‐naïve and Ate/Bev‐experienced groups.

**Results:**

Among 116 patients with advanced HCC treated with Nivo/Ipi, 57 were ICI‐naïve and 59 had prior Ate/Bev exposure. Overall objective response rate was 31.2%, higher in the ICI‐naïve group (42.6% vs. 20.0%, *p* = 0.01). However, the median duration of response was comparable between groups (24.8 vs. 23.7 months; *p* = 0.71), suggesting durable benefits regardless of prior Ate/Bev therapy. Median progression‐free survival (PFS) and overall survival (OS) were 2.5 and 11.3 months, respectively, with longer PFS (5.3 vs. 1.6 months; *p* < 0.01) and OS (16.2 vs. 7.8 months; *p* = 0.06) in the ICI‐naïve. Immune‐related adverse events (irAEs), especially thyroid dysfunction, were associated with longer PFS and OS. Notably, most Nivo/Ipi responders post‐Ate/Bev (8/11) had irAEs during Nivo/Ipi treatment, whereas no irAEs occurred during prior Ate/Bev. Nivo/Ipi responders post‐Ate/Bev revealed a high tumour mutational burden (5.71–12.75 mutations/Mb).

**Conclusion:**

Nivo/Ipi demonstrated meaningful clinical activity in patients with advanced HCC, even after Ate/Bev failure.

## Highlights

- For patients who experience disease progression after first‐line Ate/Bev, the optimal subsequent treatment strategy remains undefined.
- Nivo/Ipi demonstrated meaningful clinical activity in advanced HCC, including in a subset of patients who had previously progressed on Ate/Bev.
- Immune‐related adverse events may serve as potential predictors of clinical benefit.

**Abbreviations**
Ate/Bev: atezolizumab plus bevacizumab
CI: confidence interval
CR: complete response
CTLA‐4: cytotoxic T‐lymphocyte‐associated protein‐4
DoR: duration of response
ECOG: Eastern Cooperative Oncology Group
HCC: hepatocellular carcinoma
ICIs: immune checkpoint inhibitors
irAEs: immune‐related adverse events
Nivo/Ipi: nivolumab plus ipilimumab
ORR: objective response rate
OS: overall survival
PD: progressive disease
PD‐1: programmed death receptor‐1
PD‐L1: programmed death ligand 1
PFS: progression‐free survival
PR: partial response
SD: stable disease
TKI: tyrosine kinase inhibitor
TMB: tumour mutational burden
TRAE: treatment‐related adverse event
VEGF: vascular endothelial growth factor

## 1 Introduction

Primary liver cancer is the third leading cause of cancer‐related mortality, with hepatocellular carcinoma (HCC) accounting for approximately 90% of all cases. Most patients are diagnosed at an advanced stage, making systemic therapy the primary treatment approach. Over the past decade, immune checkpoint inhibitors (ICIs) have revolutionised the systemic treatment of HCC [1, 2, 3]. Although single‐agent ICIs, including anti‐programmed death receptor‐1 (PD‐1) and anti‐programmed death ligand 1 (PD‐L1), have failed to significantly improve overall survival (OS) with objective response rates (ORR) of 15%–20%, combination strategies have yielded more promising outcomes [4, 5, 6, 7]. These include ICIs paired with anti‐angiogenic agents or dual checkpoint blockade regimens.

The pivotal IMbrave150 trial demonstrated the superiority of atezolizumab plus bevacizumab (Ate/Bev) immunotherapy over sorafenib, leading to the practice‐changing approval of the Ate/Bev in the first‐line setting of advanced HCC [6]. Subsequently, the HIMALAYA trial also showed the superiority of the durvalumab plus tremelimumab (STRIDE regimen; Durva/Trem) combination over sorafenib [7], providing another first‐line treatment option. Most recently, the CheckMate‐9DW trial reported a significant OS benefit with the first‐line nivolumab plus ipilimumab (Nivo/Ipi) combination compared to lenvatinib or sorafenib in treatment‐naïve patients with advanced HCC [8, 9].

The Nivo/Ipi combination therapy received accelerated approval from the U.S. Food and Drug Administration in March 2020 for treating patients with HCC who had previously received sorafenib therapy based on promising results from the phase 1/2 CheckMate‐040 trial [10]. This has enabled the use of Nivo/Ipi treatment in various clinical practice settings, including patients with or without prior ICI exposure. A key question is whether different mechanisms of combination immunotherapies, including Ate/Bev [6], Durva/Trem [7] and Nivo/Ipi can be used sequentially for advanced HCC. Specifically, Ate/Bev enhances antitumor immunity by counteracting vascular endothelial growth factor (VEGF)‐mediated immunosuppression, facilitating dendritic cell maturation and increasing intratumoral T‐cell infiltration [11, 12, 13]. Meanwhile, Nivo/Ipi provides an additional antitumor response by promoting cytotoxic T‐cell activation and depleting regulatory T cells [14, 15, 16]. However, the efficacy of ICI rechallenge remains unclear, particularly following first‐line Ate/Bev failure.

Therefore, we aimed to evaluate the therapeutic efficacy and safety of Nivo/Ipi in patients with advanced HCC, stratified according to prior Ate/Bev exposure. We hypothesized that Nivo/Ipi may retain antitumor activity even after progression on Ate/Bev by engaging complementary immune mechanisms.

## 2 Methods

### 2.1 Study Population

We conducted an international multicenter retrospective study of consecutively enrolled patients with advanced HCC who were treated with Nivo/Ipi at six referral hospitals in the Republic of Korea, Hong Kong, Taiwan and Singapore (CHA Bundang Medical Centre, Haeundae Paik Hospital and Ulsan University Hospital in the Republic of Korea; Queen Mary Hospital in Hong Kong; Taipei Veterans General Hospital in Taiwan; and National Cancer Centre Singapore in Singapore) between March 2020 and December 2024. Eligible patients were those with advanced HCC diagnosed either pathologically or based on noninvasive assessment according to the American Association for the Study of Liver Diseases criteria for patients with liver cirrhosis. The inclusion criteria also required Child–Pugh class A liver function and an Eastern Cooperative Oncology Group (ECOG) performance status of 0–2. All patients received at least one dose of a Nivo/Ipi. Patients previously exposed to immunotherapy agents other than Ate/Bev were excluded. Medical records were reviewed using the institutional electronic medical record systems of each centre.

This study protocol was reviewed and approved by the institutional review board of each participating centre (CHA Bundang Medical Centre, approval No. 2024–07‐063, 2017–11‐052 and 2017–11‐054; Haeundae Paik Hospital, approval No. 2024–09‐026; Ulsan University Hospital, approval No. 2024–10‐005; Queen Mary Hospital, approval No. UW 24–714; Taipei Veterans General Hospital, TPEVGH approval No. 2023–08‐010CC; and National Cancer Centre Singapore, Singhealth Centralised approval No. 2018–3046) and was performed in accordance with the ethical standards of the institutional research committee and the recent Declaration of Helsinki. The requirement for informed consent was waived due to the retrospective design, and all patient data were anonymized and de‐identified before analysis.

### 2.2 Treatment and Assessment

Patients received nivolumab (1 mg/kg) plus ipilimumab (3 mg/kg) (N1I3), or nivolumab (3 mg/kg) plus ipilimumab (1 mg/kg) (N3I1) every 3 weeks (four doses), followed by nivolumab (240 mg) monotherapy every 2 weeks. Treatment continued until disease progression or unacceptable toxicity. The choice between N1I3 and N3I1 regimens was determined at the discretion of the treating physicians, taking into account country‐specific clinical practice patterns and regulatory considerations.

The presence of liver cirrhosis was determined based on radiologic or clinical evidence, including typical imaging findings suggestive of cirrhosis or evidence of portal hypertension, such as ascites or endoscopic documentation of oesophageal or gastric varices. Child–Pugh scores were recorded for all patients as part of routine assessment of hepatic functional reserve, irrespective of cirrhosis status.

Tumour response was assessed every 6–12 weeks according to Response Evaluation Criteria in Solid Tumour version 1.1. Radiologic evaluations were performed by local investigators at each participating centre. Responders were defined as patients who achieved complete response (CR) or partial response (PR) as the best response, while non‐responders were patients who had stable disease (SD) or progressive disease (PD) as the best response during treatment with Nivo/Ipi. Patients with disease control were categorised as those achieving CR, PR, or SD. Duration of response (DoR) was calculated from the date of the first documented CR or PR until disease progression or death, whichever occurred first. Progression‐free survival (PFS) was calculated from the date of Nivo/Ipi initiation to disease progression or death, whichever occurred first. Data of patients who were free of disease progression or loss to follow‐up were censored at the date of the last follow‐up visit. Patients who discontinued Nivo/Ipi due to its toxicity were censored at the time of the last dose injection. Overall survival was defined as the time from Nivo/Ipi initiation to death from any cause. Toxicity was evaluated according to Common Terminology Criteria for Adverse Events version 5.0, and immune‐related adverse events (irAEs) included thyroid dysfunction, adrenal insufficiency, pneumonitis and diabetes mellitus.

For the purposes of this study, ‘meaningful clinical activity’ was operationally defined as Ate/Bev‐experienced patients achieving an objective tumour response or durable disease control with clinically relevant survival outcomes from Nivo/Ipi therapy, including a duration of response exceeding 18 months or responder‐level PFS and OS comparable to those observed in ICI‐naïve patients.

### 2.3 Statistical Analysis

Categorical variables were analysed using frequencies and percentages and compared using Fisher's exact test or Pearson's chi‐square test, as appropriate. Continuous variables were analysed using the median and range and compared using a Student's *t*‐test. PFS and OS were calculated using the Kaplan–Meier method, and comparisons were made using log‐rank tests. Hazard ratios were calculated using the multivariate Cox proportional hazard model. We considered a *p*‐value < 0.05 statistically significant. Statistical analysis was performed using STATA version 18.0 (StataCorp LP, College Station, TX, USA) and R version 4.4.2.

## 3 Results

### 3.1 Baseline Characteristics

**FIGURE 1:**
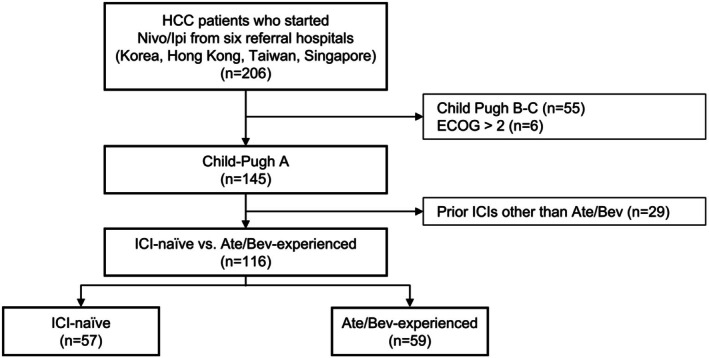
CONSORT diagram. HCC, hepatocellular carcinoma; ICI, immune checkpoint inhibitor; *n*, number.

Between March 2020 and December 2024, 206 patients with advanced HCC received Nivo/Ipi. Overall, 116 patients were included in the final analysis (Figure 1), after excluding those with Child–Pugh B–C (*n* = 55), ECOG performance status > 2 (*n* = 6) or who previously received immunotherapies rather than Ate/Bev (*n* = 29). Baseline characteristics are summarised in Table 1. The median age was 60 years (range, 18–83). The majority were male (84.5%), with an ECOG performance status of 1–2 (81.9%). Hepatitis B virus infection was the predominant aetiology of HCC (75.0%), and cirrhosis was present in 66.4% of patients. Most patients (72.4%) received Nivo/Ipi as third‐line or later systemic therapy. Regarding dosing regimens, 69.0% and 31.0% received N1I3 and N3I1, respectively.

**TABLE 1:** Baseline characteristics.

*n* (%)	Total (*n* = 116)	ICI‐naïve (*n* = 57)	Ate/Bev‐experienced (*n* = 59)	*p*
Age, median (range)	60 (18–83)	61 (18–83)	59 (33–79)	0.83
Sex				
Male	98 (84.5)	51 (89.5)	47 (79.7)	0.20
Female	18 (15.5)	6 (10.5)	12 (20.3)	
ECOG performance status				
0	21 (18.1)	14 (24.6)	7 (11.9)	0.09
1–2	95 (81.9)	43 (75.4)	52 (88.1)	
Aetiology of chronic liver disease				
Hepatitis B	87 (75.0)	40 (70.2)	47 (79.7)	0.27
Hepatitis C	7 (6.0)	6 (10.5)	1 (1.7)	
Alcohol	10 (8.6)	5 (8.8)	5 (8.5)	
MASLD/others	12 (10.3)	6 (10.5)	6 (10.2)	
Presence of cirrhosis				
No	39 (33.6)	21 (36.8)	18 (30.5)	0.47
Yes	77 (66.4)	36 (63.2)	41 (69.5)	
Child–Pugh score^a^				
5	59 (50.9)	29 (50.9)	30 (50.9)	1.00
6	57 (49.1)	28 (49.1)	29 (49.2)	
Albumin‐Bilirubin grade				
1	88 (75.9)	46 (80.7)	42 (71.2)	0.23
2	28 (24.1)	11 (19.3)	17 (28.8)	
BCLC stage				
B	19 (16.4)	14 (24.6)	5 (8.5)	0.02
C	97 (83.6)	43 (75.4)	54 (91.5)	
AFP (ng/mL) (*n* = 115)				
< 400	57 (49.6)	29 (50.9)	28 (48.3)	0.78
≥ 400	58 (50.4)	28 (49.1)	30 (51.7)	
Extrahepatic metastasis	92 (79.3)	41 (71.9)	51 (86.4)	0.07
Presence of PVTT	30 (25.9)	18 (31.6)	12 (20.3)	0.21
Number of prior systemic treatment				
< 2	32 (27.6)	29 (50.9)	3 (5.1)	< 0.01
≥ 2	84 (72.4)	28 (49.1)	56 (94.9)	
Treatment dose of Nivo/Ipi				
Nivo 1 mg/kg + Ipi 3 mg/kg	80 (69.0)	34 (59.7)	46 (78.0)	0.03
Nivo 3 mg/kg + Ipi 1 mg/kg	36 (31.0)	23 (40.4)	13 (22.0)	

Abbreviations: AFP, alpha‐fetoprotein; BCLC, Barcelona Clinic Liver Cancer; ECOG, Eastern Cooperative Oncology Group; ICI, immune checkpoint inhibitor; MASLD, metabolic dysfunction‐ associated steatotic liver disease; n, number; PVTT, portal vein tumour thrombosis.

^a^ Child–Pugh scores were recorded for all patients as an assessment of liver functional reserve, irrespective of radiologic clinical evidence of cirrhosis.

Patients were stratified into two groups based on prior exposure to Ate/Bev: immunotherapy‐naïve (ICI‐naïve, *n* = 57) and Ate/Bev‐experienced (*n* = 59). All patients in the Ate/Bev‐experienced group had documented disease progression on prior Ate/Bev therapy. Among the ICI‐naïve patients, those who received Nivo/Ipi as second‐ or later‐line therapy had previously received multi‐kinase inhibitors such as sorafenib, lenvatinib, regorafenib, or cabozantinib. Detailed treatment history is provided in Table S1. Compared to the ICI‐naïve group, the Ate/Bev‐experienced group had a significantly higher proportion of BCLC stage C (91.5% vs. 75.4%, *p* = 0.02), prior exposure to two or more lines of systemic treatment (94.9% vs. 49.1%, *p* < 0.01) and receipt of N1I3 regimen (78.0% vs. 59.7%, *p* = 0.03). Other baseline characteristics were generally comparable between the two groups.

### 3.2 Treatment Outcomes

**FIGURE 2:**
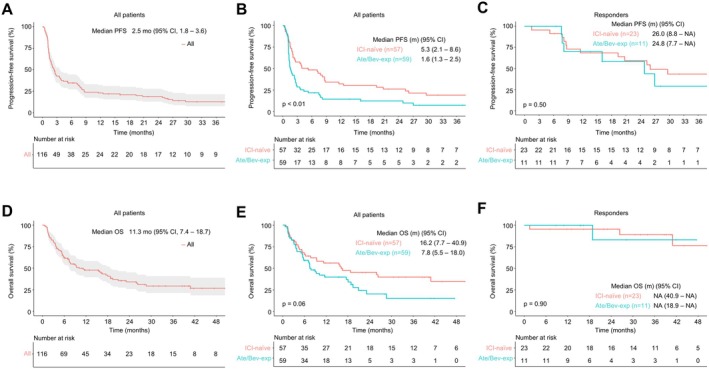
Survival outcomes. (A) Progression‐free survival (PFS) in all patients. (B) PFS according to prior Atezolizumab/Bevacizumab (Ate/Bev) experience. (C) PFS of Nivolumab/Ipilimumab (Nivo/Ipi) responders according to prior Ate/Bev experience. (D) Overall survival (OS) in all patients. (E) OS according to prior Ate/Bev experience. (F) OS of Nivo/Ipi responders according to prior Ate/Bev experience. CI, confidence interval; m, months; NR, not reached.

At a median follow‐up duration of 34.5 months (95% confidence interval (CI), 25.9–39.3), the ORR among 109 evaluable patients was 31.2%, including 6 CRs (5.5%) and 28 PRs (25.7%). Disease control was achieved in 45.9% of patients (Table 2). The median DoR was 24.8 months (95% CI, 12.4–not reached) (Figure S1A). Seven patients lacked post‐baseline imaging and were therefore excluded from response evaluation due to early clinical deterioration, treatment discontinuation, or death. Median PFS was 2.5 months (95% CI, 1.8–3.6) and median OS was 11.3 months (95% CI, 7.4–18.7) (Figure 2A,D).

**TABLE 2:** Response to nivolumab plus ipilimumab treatment (109 evaluable patients).

	All patients (*n* = 109)	ICI‐naïve (*n* = 54)	Ate/Bev‐experienced (*n* = 55)	*p*
Best response to Nivo/Ipi, *n* (%)				0.04
Complete response	6 (5.5)	4 (7.4)	2 (3.6)	
Partial response	28 (25.7)	19 (35.2)	9 (16.4)	
Stable disease	16 (14.7)	9 (16.7)	7 (12.7)	
Progressive disease	59 (54.1)	22 (40.7)	37 (67.3)	
Objective response rate, *n* (%)	34 (31.2)	23 (42.6)	11 (20.0)	0.01
Disease control rate, *n* (%)	50 (45.9)	32 (59.3)	18 (32.7)	< 0.01

Abbreviations: ICI, immune checkpoint inhibitor; *n*, number.

Subgroup analyses revealed significantly better efficacy outcomes in the ICI‐naïve group than in the Ate/Bev‐experienced group. The ORR was 42.6% in ICI‐naïve patients versus. 20.0% in the Ate/Bev‐experienced group (*p* = 0.01) and DCR was 59.3% versus. 32.7%, respectively (*p* < 0.01) (Table 2). However, DoR remained comparable between groups (24.8 vs. 23.7 months; *p* = 0.71) (Figure S1B).

The median PFS was longer in the ICI‐naïve group (5.3 vs. 1.6 months; *p* < 0.01), and the median OS was numerically higher (16.2 vs. 7.8 months; *p* = 0.06) (Figure 2B,E). Among patients who achieved a response to Nivo/Ipi, survival outcomes were comparable irrespective of prior Ate/Bev treatment (Figure 2C,F). No cases of pseudoprogression or hyperprogression were identified during response evaluation. An exploratory subgroup analysis comparing the two dosing regimens (N1I3 vs. N3I1) showed no significant differences in ORR, PFS and OS (Table S2 and Figure S2).

### 3.3 Treatment‐Related Adverse Events (TRAEs)

Among the 116 patients treated with Nivo/Ipi, the most frequent TRAE of any grade was hepatitis (36.2%), followed by pruritus (21.6%) and hyperbilirubinemia (20.7%). Grade ≥ 3 TRAEs included hepatitis (11.2%), hyperbilirubinemia (5.2%), pruritis, rash and lipase increase (each of them in 2.6%). Of common irAEs of interest, adrenal insufficiency (8.6%), pneumonitis (7.8%), and hypothyroidism (6.9%) were observed. Grade ≥ 3 irAEs included pneumonitis (3.4%) and adrenal insufficiency (1.7%) (Table 3).

**TABLE 3:** Treatment‐related adverse events.

*n* (%)	Total (*n* = 116)		ICI‐naïve (*n* = 57)		Ate/Bev‐experienced (*n* = 59)	
	Any grade	Grade ≥ 3	Any grade	Grade ≥ 3	Any grade	Grade ≥ 3
Hepatitis	42 (36.2)	13 (11.2)	21 (36.8)	6 (10.5)	21 (35.6)	7 (11.9)
Pruritus	25 (21.6)	3 (2.6)	12 (21.1)	1 (1.8)	13 (22.0)	2 (3.4)
Hyperbilirubinemia	24 (20.7)	6 (5.2)	12 (21.1)	3 (5.3)	12 (20.3)	3 (5.1)
Anaemia	20 (17.2)	2 (1.7)	6 (10.5)	1 (1.8)	14 (23.7)	1 (1.7)
Rash	18 (15.5)	3 (2.6)	8 (14.0)		10 (16.9)	3 (5.1)
Fatigue	18 (15.5)	1 (0.9)	11 (19.3)		7 (11.9)	1 (1.7)
Neutropenia	15 (12.9)	4 (3.4)	8 (14.0)	2 (3.5)	7 (11.9)	2 (3.4)
Thrombocytopenia	13 (11.2)		6 (10.5)		7 (11.9)	
Adrenal insufficiency	10 (8.6)	2 (1.7)	7 (12.3)	1 (1.8)	3 (5.1)	1 (1.7)
Nausea	9 (7.8)	1 (0.9)	6 (10.5)		3 (5.1)	1 (1.7)
Pneumonitis	9 (7.8)	4 (3.4)	5 (8.8)	2 (3.5)	4 (6.8)	2 (3.4)
Diarrhoea	9 (7.8)	2 (1.7)	7 (12.3)	1 (1.8)	2 (3.4)	1 (1.7)
Hypothyroidism	8 (6.9)		5 (8.8)		3 (5.1)	
Anorexia	7 (6.0)	1 (0.9)	4 (7.0)		3 (5.1)	1 (1.7)
Lipase increase	5 (4.3)	3 (2.6)	3 (5.3)	2 (3.5)	2 (3.4)	1 (1.7)
Diabetes mellitus	4 (3.4)	1 (0.9)	2 (3.5)	1 (1.8)	2 (3.4)	
Hyperthyroidism	3 (2.6)	1 (0.9)	3 (5.3)	1 (1.8)		
Vomiting	2 (1.7)		2 (3.5)			

Abbreviations: ICI, immune checkpoint inhibitor; *n*, number.

When comparing safety profiles between subgroups, the overall incidence of TRAEs was similar between ICI‐naïve and Ate/Bev‐experienced patients. However, irAEs tended to occur more frequently in the ICI‐naïve group, particularly endocrine‐related events such as thyroid dysfunction and adrenal insufficiency. Additionally, the incidence of TRAE tended to be higher in the N1I3 group than N3I1 group (Table S3).

Among the Ate/Bev‐experienced group, data on prior irAEs were available for all but 17 patients. The presence of irAEs during prior Ate/Bev therapy did not show a significant correlation with the occurrence of irAEs upon subsequent Nivo/Ipi treatment (Table S4). The median treatment duration was 60 days (range, 12–1623+ days) in patients without irAEs and 484 days (range, 8–1639+ days) in those who developed irAEs. Most irAEs developed relatively early during treatment (median time to onset, 58 days; range, 5–411 days), including thyroid dysfunction (median, 70 days; range, 30–259 days). Nevertheless, longer treatment exposure among responders may contribute to a higher cumulative incidence of immune‐related toxicities.

**FIGURE 3:**
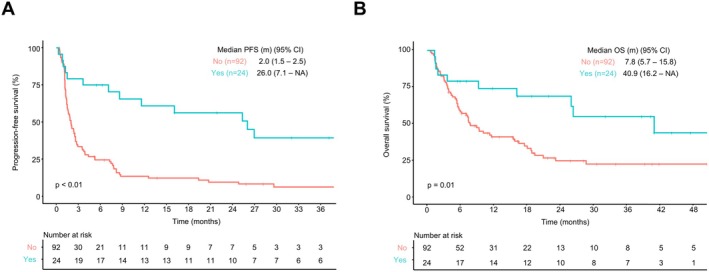
Survival outcomes according to the occurrence of immune‐related adverse events (irAEs). (A) Overall survival. (B) Progression‐free survival. *The following irAEs of any grades were included for this analysis: adrenal insufficiency, hypothyroidism, pneumonitis, diabetes mellitus and hyperthyroidism.

The occurrence of irAEs was significantly associated with favourable outcomes. Patients with irAEs had significantly longer PFS (*p* < 0.01) and OS than those without irAEs (*p* = 0.01) (Figure 3). Subgroup analyses revealed that thyroid dysfunction was associated with improved PFS and OS across all groups, regardless of prior Ate/Bev exposure (Figure S3). Similarly, adrenal insufficiency was linked to prolonged PFS in both ICI‐naïve and Ate/Bev‐experienced patients; however, OS benefit did not reach statistical significance (Figure S4). Moreover, the median time to onset of overall irAEs and thyroid dysfunction were 58 days (range, 5–411 days) and 70 days (range, 30–259 days), respectively.

### 3.4 Characteristics of Nivo/Ipi Responder After Failure of Prior Ate/Bev

**FIGURE 4:**
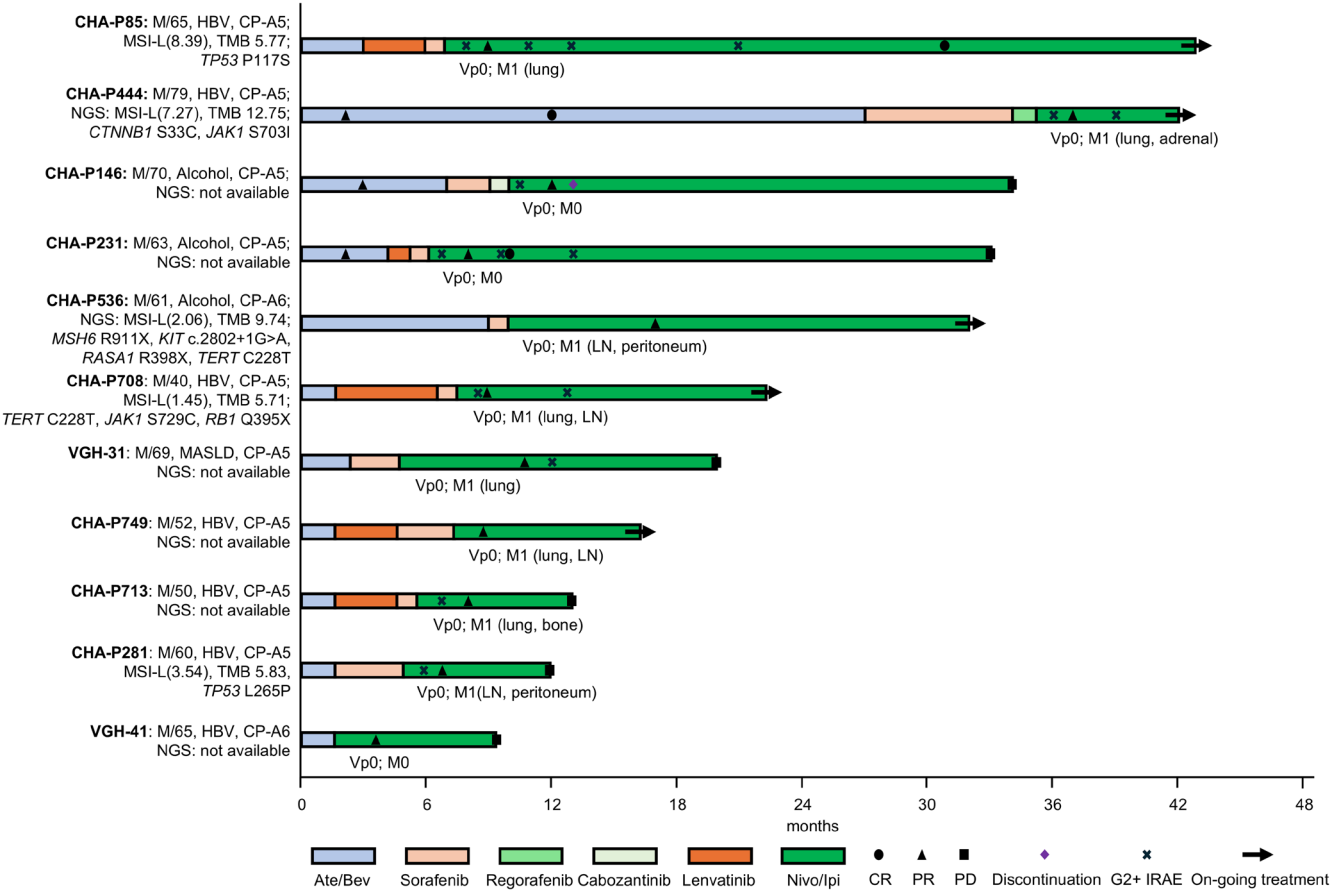
Disease courses and clinicogenetic summary of Nivo/Ipi responders after failure of prior Ate/Bev. CP–A, Child‐Pugh class A; CR, complete response; PD, progressive disease; PR, partial response.

Among the 55 patients previously treated with Ate/Bev, 11 (20.0%) achieved an objective response to subsequent Nivo/Ipi. Their clinical and molecular characteristics are summarised in Table 4 and Figure 4, respectively.

**TABLE 4:** Characteristics of Atezolizumab/Bevacizumab‐experienced group (evaluable).

*n* (%)	Total (*n* = 55)	Non‐responder (*n* = 44)	Responder (*n* = 11)	*p*
Age, median (range)	59 (33–79)	59 (33–77)	63 (40–79)	0.15
Sex				
Male	44 (80.0)	33 (75.0)	11 (100.0)	0.10
Female	11 (20.0)	11 (25.0)	0	
ECOG status				
0	7 (12.7)	7 (15.9)	0	0.32
1–2	48 (87.3)	37 (84.1)	11 (100.0)	
Aetiology of chronic liver disease				
Hepatitis B	44 (80.0)	37 (84.1)	7 (63.6)	0.17
Hepatitis C	1 (1.8)	1 (2.3)	0	
Alcohol	5 (9.1)	2 (4.6)	3 (27.3)	
MASLD/Others	5 (9.1)	4 (9.1)	1 (9.1)	
Child‐Pugh score^a^				
5	28 (50.9)	19 (43.2)	9 (81.8)	0.04
6	27 (49.1)	25 (56.8)	2 (18.2)	
Albumin‐bilirubin grade				
1	41 (74.6)	31 (70.5)	10 (90.9)	0.26
2	14 (25.5)	13 (29.6)	1 (9.1)	
BCLC stage				
B	4 (7.3)	2 (4.6)	2 (18.2)	0.18
C	51 (92.7)	42 (95.5)	9 (81.2)	
AFP (ng/mL) (*n* = 70)				
< 400	28 (51.9)	22 (51.2)	6 (54.6)	1.00
≥ 400	26 (48.2)	21 (48.8)	5 (45.5)	
Extrahepatic metastasis	48 (87.3)	40 (90.9)	8 (72.7)	0.13
Presence of PVTT	12 (21.8)	12 (27.3)	0	0.10
Extent of intrahepatic lesion(s)				
< 50%	42 (76.4)	31 (70.5)	11 (100.0)	0.05
≥ 50%	13 (23.6)	13 (29.6)	0	
Number of prior systemic treatment				
< 2	3 (5.5)	2 (4.6)	1 (9.1)	0.50
≥ 2	52 (94.6)	42 (95.5)	10 (90.9)	
Best response to prior Ate/Bev				
CR/PR	7 (12.7)	4 (9.1)	3 (27.3)	0.29
SD	18 (32.7)	16 (36.4)	2 (18.2)	
PD	28 (50.9)	22 (50.0)	6 (54.6)	
Unknown	2 (3.6)	2 (4.6)	0	
Treatment dose of Nivo/Ipi				
Nivo 1 mg/kg + Ipi 3 mg/kg	44 (80.0)	35 (79.6)	9 (81.8)	1.00
Nivo 3 mg/kg + Ipi 1 mg/kg	11 (20.0)	9 (20.5)	2 (18.2)	
irAEs during prior Ate/Bev treatment				
No	36 (65.5)	27 (61.4)	9 (81.8)	0.66
Yes	2 (3.6)	2 (4.6)	0	
Unknown	17 (30.9)	15 (34.1)	2 (18.2)	
Prior exposure to multi‐kinase inhibitors^b^				
Sorafenib	43 (78.2)	33 (75.0)	10 (90.9)	0.42
Lenvatinib	26 (47.3)	21 (47.7)	5 (45.5)	1.00
Regorafenib	7 (12.7)	6 (13.6)	1 (9.1)	1.00
Cabozantinib	10 (10.2)	9 (20.5)	1 (9.1)	0.67

Abbreviations: AFP, alpha‐fetoprotein; BCLC; Barcelona Clinic Liver Cancer; CR, complete response; ECOG, Eastern Cooperative Oncology Group; irAE, immune‐related adverse events; MASLD, metabolic dysfunction‐associated steatotic liver disease; n, number; PD, progressive disease; PR, partial response; PVTT, portal vein tumour thrombosis.

^a^ Child–Pugh scores were recorded for all patients as an assessment of liver functional reserve, irrespective of radiologic clinical evidence of cirrhosis.

^b^ Percentages within each group may exceed 100% because some patients received more than one multi‐kinase inhibitor sequentially.

Of the 11 responders, three (27.3%) had also responded to prior Ate/Bev (CR or PR), with a median treatment duration of 2.0 months (range, 1–27 months). Ten received at least one tyrosine kinase inhibitor (TKI) between Ate/Bev and Nivo/Ipi, with a median interval of 4.5 months (range, 2.1–15.7). At data cutoff, five responders remained on Nivo/Ipi treatment without disease progression.

Among the 11 responders, most had better‐preserved liver function, with nine patients having a Child–Pugh score of 5. None had evidence of portal vein tumour thrombosis (PVTT), and all had an intrahepatic tumour burden of less than 50%. Notably, most of them (10/11) previously received two or more lines of systemic treatment. The majority of them (8/11) experienced irAEs during Nivo/Ipi therapy, whereas none of them had experienced irAEs during prior Ate/Bev treatment.

Next‐generation sequencing data were available for five of the 11 responders. Despite no consistent genetic alterations, all five exhibited relatively high tumour mutational burden (TMB), ranging from 5.71 to 12.75 mutations/Mb, exceeding the historical average TMB for HCC (approximately 1.4 mutations/Mb) [17]. Mutations in *TP53*, *CTNNB1*, *MSH6*, *JAK1* and *RB1* were detected in individual cases. A responder with *MSH6* R911X mutation, suggesting a potential mismatch repair‐deficient phenotype, achieved a PFS of 9 months on Ate/Bev and maintained a durable response exceeding 24 months on Nivo/Ipi. Two responders carried the *TERT* C228T mutation, which is prevalent in HCC [18]. Notably, one responder harboured a *CTNNB1* mutation, which is traditionally associated with immune exclusion [19], but showed sustained responses to both Ate/Bev and Nivo/Ipi.

## 4 Discussion

This multicenter study provides real‐world evidence across Asia regarding the efficacy of Nivo/Ipi in patients with advanced HCC who have previously been treated with Ate/Bev. While the landscape of frontline ICI‐based therapies continues to evolve, the optimal subsequent strategies following first‐line Ate/Bev remains undefined. Although TKIs are commonly used in this setting, their efficacy is frequently limited by modest and short‐lived responses. Our findings suggest that Nivo/Ipi retains an antitumor activity in selected patients despite prior Ate/Bev exposure. While there is growing interest in the complementary roles of different immunotherapeutic regimens, clear evidence guiding optimal sequencing and patient selection criteria remains scarce. Our results provide valuable insights into this evolving landscape.

Although the Ate/Bev‐experienced group was more heavily pretreated and exhibited a lower ORR, PFS and OS among responders were comparable regardless of prior Ate/Bev exposure, suggesting that anti–PD‐1/anti–CTLA‐4 therapy retains efficacy through distinct or complementary immune mechanisms, even after failure of anti–PD‐L1/anti–VEGF therapy. One potential explanation is that anti–CTLA‐4 agents such as ipilimumab exert immunologic effects that are distinct from those of anti–PD‐L1/VEGF therapy. Specifically, anti–CTLA‐4 therapy can deplete intratumoral regulatory T cells and promote de novo T cell priming [20], thereby reversing liver‐specific immune tolerance—a key immunologic barrier in HCC. These mechanisms may restore intrahepatic immune responsiveness and enhance antitumor activity in settings where anti–PD‐L1/anti–VEGF combinations have failed. The observation that a subset of Ate/Bev‐experienced responders achieved durable disease control with survival outcomes similar to those of ICI‐naïve responders supports the hypothesis that sequential immunotherapy using mechanistically non‐redundant combinations may provide meaningful clinical benefit in selected patients. Although these observations are derived from a limited number of responders, they offer important insights into the potential benefit of a salvage strategy after Ate/Bev failure. Interestingly, a dichotomous response pattern was observed in the Ate/Bev‐experienced group, with patients either deriving substantial benefits or experiencing rapid disease progression. This ‘all‐or‐none’ phenomenon reflects the immunologic heterogeneity of HCC and highlights the potential utility of Nivo/Ipi as a salvage strategy in biomarker‐enriched populations [9].

Another significant observation from our study is the association between irAEs and improved clinical outcomes. Patients with endocrine toxicity—particularly thyroid dysfunction—experienced significantly longer PFS and OS compared to those without irAEs. This aligns with prior studies suggesting that irAEs serve as indicators of effective immune activation [12, 21, 22]. Importantly, this association was observed across both ICI‐naïve and Ate/Bev‐experienced patients, although thyroid‐related irAEs were more frequently reported in the ICI‐naïve subgroup. Moreover, among patients who responded to Nivo/Ipi after failing Ate/Bev, most responders had irAEs during Nivo/Ipi therapy, whereas none had experienced such events during prior Ate/Bev treatment. These findings suggest that the emergence of irAEs serves as a useful on‐treatment indicator for identifying patients more likely to benefit from ongoing ICI therapy. However, as another common endocrine toxicity—adrenal insufficiency—showed benefit only in PFS, further studies are warranted to elucidate the difference.

Despite the limited sample size, several clinical and molecular features appeared to be potentially associated with objective response to Nivo/Ipi following prior Ate/Bev failure. Patients without PVTT, with a low intrahepatic tumour burden (< 50%) and preserved liver function tended to experience more favourable responses. In addition, relatively higher TMB levels (range, 5.71–12.75 mutations/Mb), exceeding the reported average for HCC (1.4 mutations/Mb) [17] and the occurrence of irAEs were more frequently observed among responders. Notably, one patient harbouring a *CTNNB1* mutation, traditionally considered a poor response to ICIs [19, 23], experienced durable responses to both Ate/Bev and Nivo/Ipi, highlighting complexity and context dependence of predictive biomarkers.

These observations also suggest that tumour biology may differ between the ICI‐naïve and Ate/Bev‐experienced groups. As most patients in the Ate/Bev‐experienced group received Nivo/Ipi as third‐or later‐line therapy, inherent imbalances in clinical status, treatment burden, and tumour biology are unavoidable. This cohort tended to present with more advanced disease, which may partly account for the observed differences in treatment outcomes. Such variations in underlying tumour‐intrinsic and host‐related factors should be considered when interpreting the efficacy of sequential immunotherapy. Accordingly, direct comparisons between these groups should be regarded as exploratory, given the potential imbalances in treatment lines. Prospective studies in biologically homogeneous populations are warranted to validate these findings.

This study has several limitations. First, the retrospective design and relatively small sample size limit the generalizability of our findings. Accordingly, the results should be interpreted as hypothesis‐generating rather than confirmatory. Second, as all patients were of East Asian ethnicity and the study was conducted in hepatitis B virus‐endemic regions, further investigation is warranted to determine the applicability of these results in the global population. Third, radiologic assessments were investigator‐based without central reviews, and the heterogeneous imaging intervals may have affected the consistency of evaluations. Lastly, the lack of comprehensive molecular profiling for all patients limits our ability to elucidate the biological mechanisms underlying treatment response or resistance to Nivo/Ipi.

In conclusion, Nivo/Ipi demonstrated meaningful clinical activity in advanced HCC, including in a subset of patients who had previously progressed on Ate/Bev. These findings support the potential role of sequential immunotherapy using mechanistically distinct agents and underscore the need for biomarker‐driven approaches to guide patient selection and optimise treatment sequencing in this population.

## Author Contributions

Conceived and designed the analysis: Kim J.S., Wong J., Chen S‐C., Yang H., Kim C., Yau T. and Chon H.J. Collected the data: Kim J.S., Wong J., Chen S.C., Yang H., Kim Y., Kang B., Kim I., Kim H., An C., Jang S.J., Tai D., Lim H.Y., Kim C., Yau T. and Chon H.J. Contributed data or analysis tools: Kim J.S., Wong J., Yang H., Kim Y., Tai D., Kudo M., Lim H.Y., Kim C., Yau T. and Chon H.J. Performed the analysis: Kim J.S., Wong J., Chen S.C., Yang H., Kim Y., Kim C., Yau T. and Chon H.J. Wrote the paper: Kim J.S., Wong J., Chen S.C., Yang H., Tai D., Kudo M., Kim C., Yau T. and Chon H.J.

## Funding

This study was supported by National Research Foundation of Korea, NRF‐2023R1A2C2004339.

## Conflicts of Interest

Hong Jae Chon consults for Eisai, Roche, Bayer, ONO, MSD, BMS, Sanofi, Servier, AstraZeneca, SillaJen, Menarini and GreenCross Cell; and has research grants from Roche, Dong‐A ST and Boryung Pharmaceuticals. Chan Kim consults for Roche, ONO, MSD, BMS, Oncocross and Virocure and receives research funding from Boryung Pharmaceuticals, Oncocross, SillaJen and Virocure. Masatoshi Kudo consults for ONO, Chugai, Roche, Eisai and AstraZeneca; and received a lecture fee from Chugai, Eisai, AstraZeneca; and has a research grant from ONO, Chugai, Roche, Eisai, AstraZeneca.

## Supporting information




**Figure S1:** Duration of response for nivolumab plus ipilimumab responders.
**Figure S2:** Survival outcomes according to dosing regimen of nivolumab plus ipilimumab.
**Figure S3:** Survival outcomes according to presence of immune‐related thyroid dysfunction.
**Figure S4:** Survival outcomes according to presence of immune‐related adrenal insufficiency.
**Table S1:** Distribution of prior exposure to multi‐kinase inhibitors in ICI‐naïve group (*n* = 57)a.
**Table S2:** Response to nivolumab plus ipilimumab treatment according to dosing regimen (109 evaluable patients).
**Table S3:** Treatment‐related adverse events according to dosing regimen.
**Table S4:** Correlation between the occurrence of immune‐related adverse events (irAEs) during prior atezolizumab plus bevacizumab therapy and subsequent nivolumab plus ipilimumab treatment.

## Data Availability

The data that supports the findings of this study are available in the Supporting Information of this article.
