# Supplementary material for: Outcomes of Nivolumab Plus Ipilimumab After Atezolizumab Plus Bevacizumab in Advanced HCC: An International Multicentre Study
Source: Liver Int. 2025 Dec 26;46(2):e70493. doi: 10.1111/liv.70493 (PMC12742031; doi:10.1111/liv.70493)
Supplement: Supplementary file 1 — Figure S1: Duration of response for nivolumab plus ipilimumab responders. Figure S2: Survival outcomes according to dosing regimen of nivolumab plus ipilimumab. Figure S3: Survival outcomes according to presence of immune‐related thyroid dysfunction. Figure S4: Survival outcomes according to presence of immune‐related adrenal insufficiency. Table S1: Distribution of prior exposure to multi‐kinase inhibitors in ICI‐naïve group (n = 57)a. Table S2: Response to nivolumab plus ipilimumab treatment according to dosing regimen (109 evaluable patients). Table S3: Treatment‐related adverse events according to dosing regimen. Table S4: Correlation between the occurrence of immune‐related adverse events (irAEs) during prior atezolizumab plus bevacizumab therapy and subsequent nivolumab plus ipilimumab treatment. [file LIV-46-0-s001.docx]

**Supplementary Materials**

**
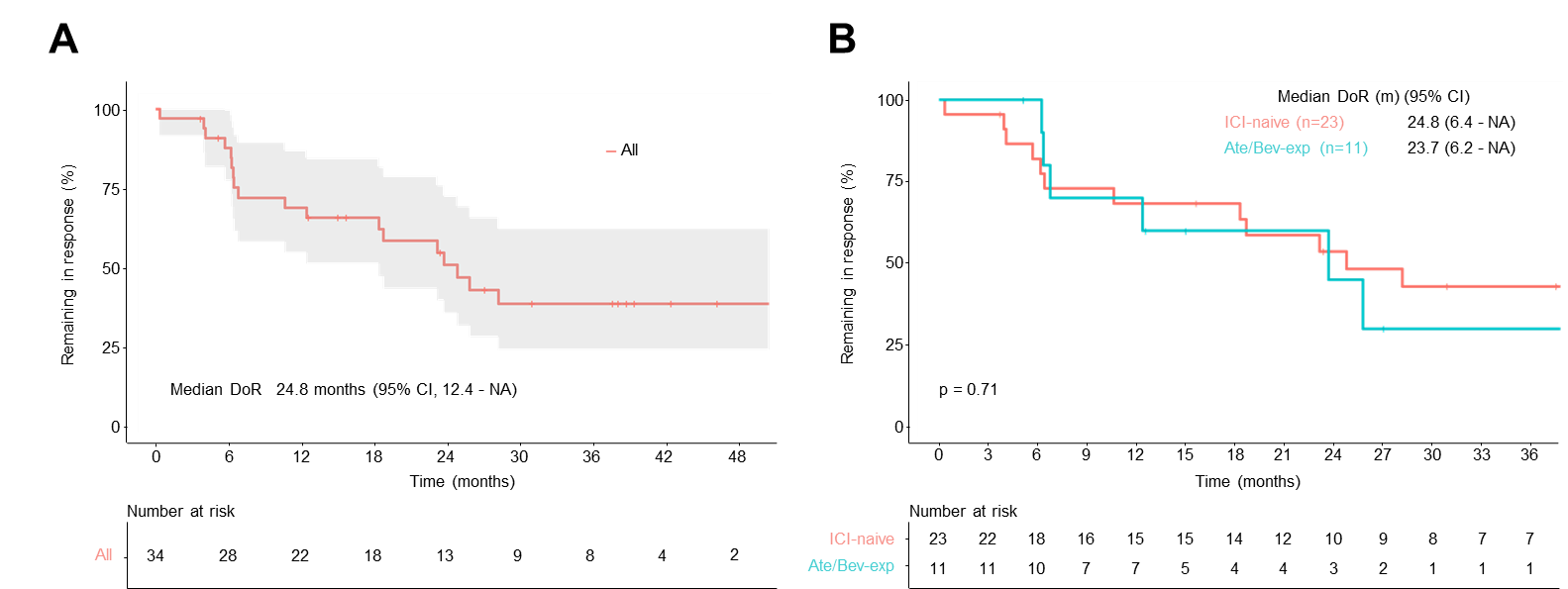
**

**Supplementary Figure 1. Duration of response for nivolumab plus ipilimumab responders.**

A. Overall population, B. According to prior exposure to atezolizumab plus bevacizumab.


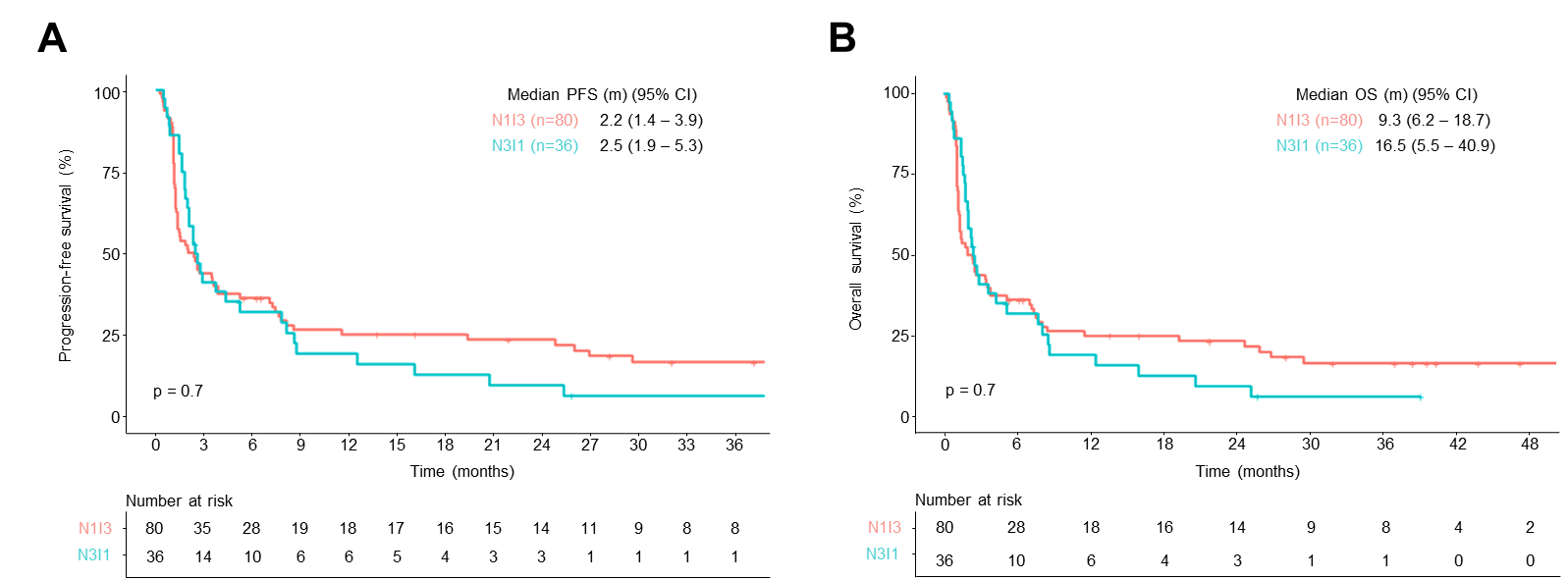


**Supplementary Figure 2. Survival outcomes according to dosing regimen of nivolumab plus ipilimumab**

A. Progression-free survival, B. Overall survival (OS). Abbreviations: N1I3, Nivolumab (1 mg/kg) plus Ipilimumab (3 mg/kg) every 3 weeks (four doses), followed by nivolumab (240 mg) monotherapy every 2 weeks; N3I1, Nivolumab (3 mg/kg) plus Ipilimumab (1 mg/kg) every 3 weeks (four doses), followed by nivolumab (240 mg) monotherapy every 2 weeks.


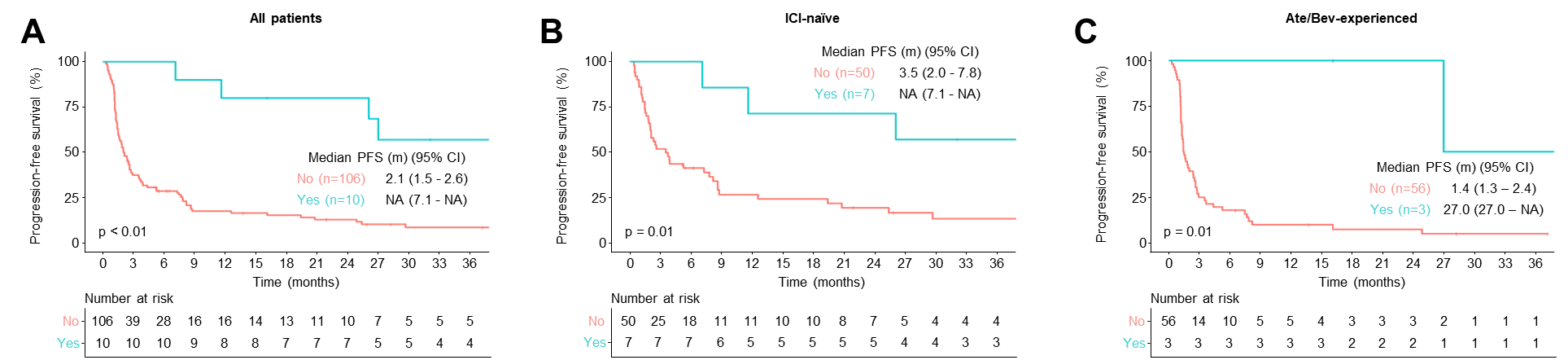


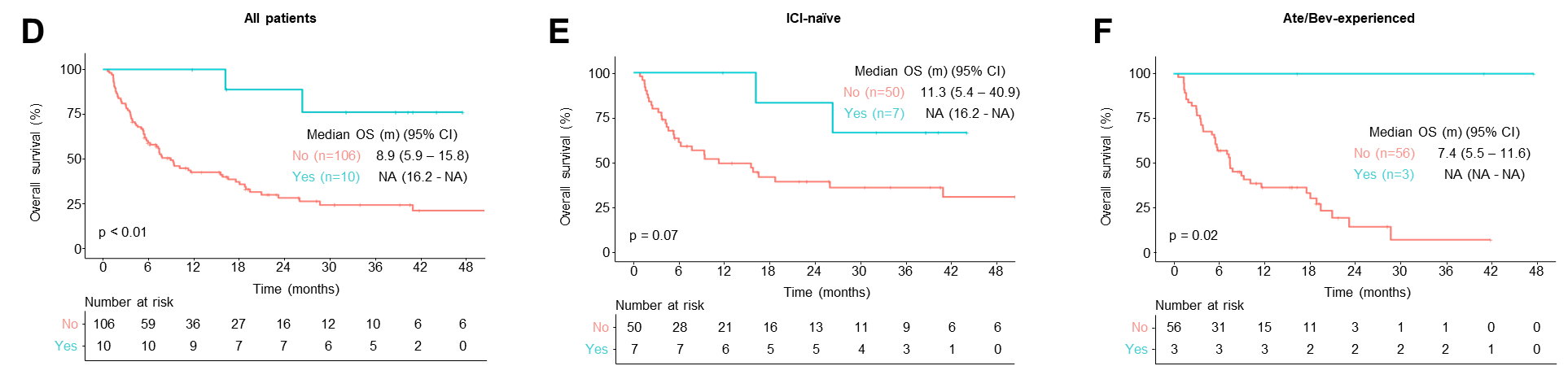


**Supplementary Figure 3. Survival outcomes according to presence of immune-related thyroid dysfunction**

Progression-free survival (PFS) in A. All patients, B. Immune-checkpoint inhibitor (ICI)-naïve group, C. Ate/Bev-experienced group; Overall survival (OS) in D. All patients, E. ICI-naïve group, F. Ate/Bev-experienced group.

**
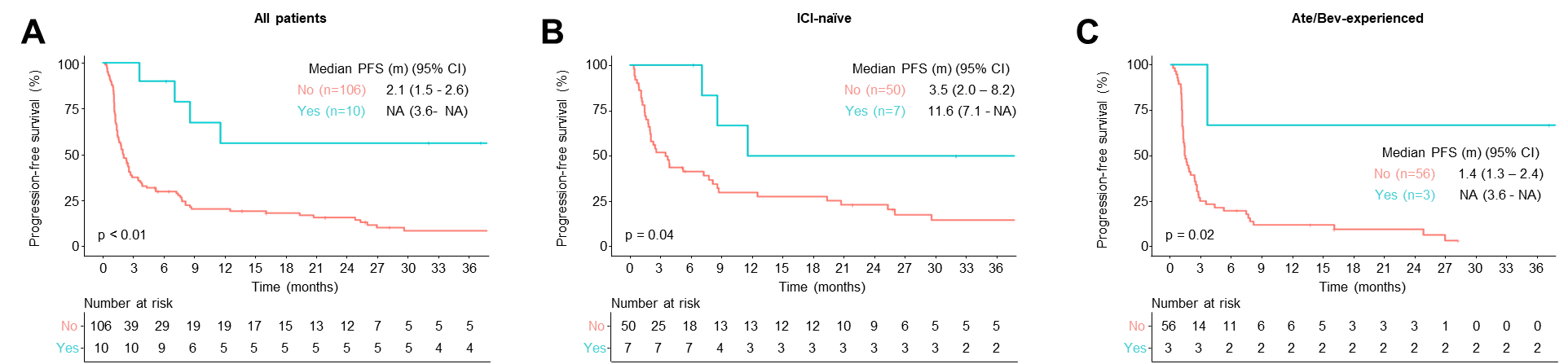
**

**
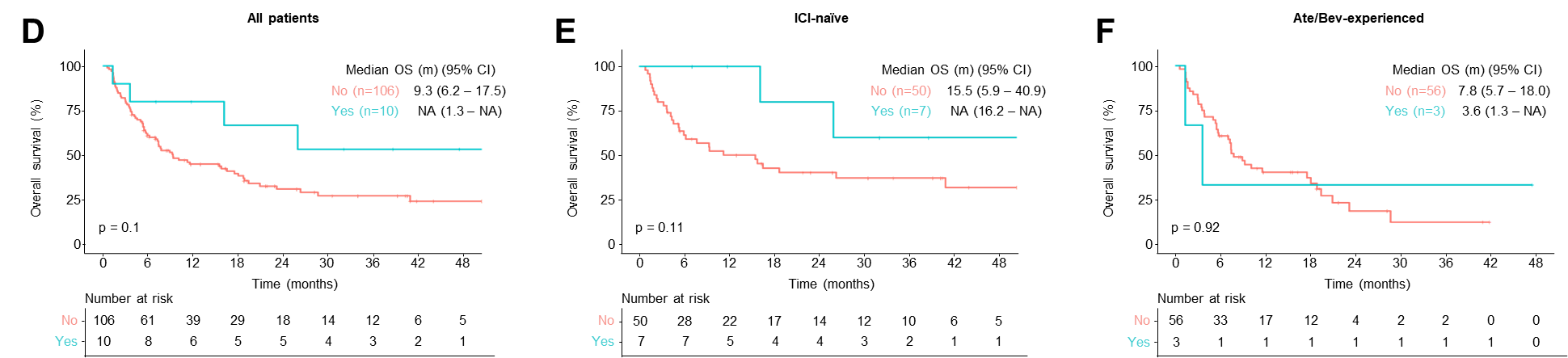
**

**Supplementary Figure 4. Survival outcomes according to presence of immune-related adrenal insufficiency**

Progression-free survival (PFS) in A. All patients, B. Immune-checkpoint inhibitor (ICI)-naïve group, C. Ate/Bev-experienced group; Overall survival (OS) in D. All patients, E. ICI-naïve group, F. Ate/Bev-experienced group.

**Supplementary Table 1. Distribution of prior exposure to multi-kinase inhibitors in ICI-naïve group (n = 57)^a^**

| **Types of multi-kinase inhibitors** | **Number of patients (%)** |
| --- | --- |
| None^a^ | 16 (28.1) |
| Sorafenib | 31 (54.4) |
| Lenvatinib | 23 (40.4) |
| Regorafenib | 13 (22.8) |
| Cabozantinib | 2 (3.5) |

^a^Sixteen patients received nivolumab plus ipilimumab in the first-line setting

**Supplementary Table 2. Response to nivolumab plus ipilimumab treatment according to dosing regimen (109 evaluable patients)**

|  | **All patients (n=109)** | **N1I3^a^ (n=77)** | **N3I1^b^ (n=32)** | **P-value** |
| --- | --- | --- | --- | --- |
| **Best response to Nivo/Ipi, n (%)** |  |  |  | 0.94 |
| Complete response | 6 (5.5) | 5 (6.5) | 1 (3.1) |  |
| Partial response | 28 (25.7) | 19 (24.7) | 9 (28.1) |  |
| Stable disease | 16 (14.7) | 11 (14.3) | 5 (15.6) |  |
| Progressive disease | 59 (54.1) | 42 (54.6) | 17 (53.1) |  |
| **Objective response rate, n (%)** | 34 (31.2) | 24 (31.2) | 10 (31.3) | 0.99 |
| **Disease control rate, n (%)** | 50 (45.9) | 35 (45.5) | 15 (46.9) | 0.89 |

^a^ Nivolumab (1 mg/kg) plus Ipilimumab (3 mg/kg) every 3 weeks (four doses), followed by nivolumab (240 mg) monotherapy every 2 weeks.

^b^ Nivolumab (3 mg/kg) plus Ipilimumab (1 mg/kg) every 3 weeks (four doses), followed by nivolumab (240 mg) monotherapy every 2 weeks.

**Supplementary Table 3. Treatment-related adverse events according to dosing regimen**

| **n (%)** | **Total (n=116)** | | **N1I3^a^ (n=80)** | | **N3I1^b^ (n=36)** | |
| --- | --- | --- | --- | --- | --- | --- |
|  | **Any grade** | **Grade ≥3** | **Any grade** | **Grade ≥3** | **Any grade** | **Grade ≥3** |
| Hepatitis | 42 (36.2) | 13 (11.2) | 40 (50.0) | 13 (16.3) | 2 (5.6) |  |
| Pruritus | 25 (21.6) | 3 (2.6) | 23 (28.8) | 3 (3.8) | 2 (5.6) |  |
| Hyperbilirubinemia | 24 (20.7) | 6 (5.2) | 23 (28.8) | 6 (7.5) | 1 (2.8) |  |
| Anemia | 20 (17.2) | 2 (1.7) | 20 (25.0) | 2 (2.5) |  |  |
| Rash | 18 (15.5) | 3 (2.6) | 14 (17.5) | 3 (3.8) | 4 (11.1) |  |
| Fatigue | 18 (15.5) | 1 (0.9) | 16 (20.0) | 1 (1.3) | 2 (5.6) |  |
| Neutropenia | 15 (12.9) | 4 (3.4) | 15 (18.8) | 4 (5.0) |  |  |
| Thrombocytopenia | 13 (11.2) |  | 13 (16.3) |  |  |  |
| Adrenal insufficiency | 10 (8.6) | 2 (1.7) | 10 (12.5) | 2 (2.5) |  |  |
| Nausea | 9 (7.8) | 1 (0.9) | 9 (11.3) | 1 (1.3) |  |  |
| Pneumonitis | 9 (7.8) | 4 (3.4) | 6 (7.5) | 4 (5.0) | 3 (8.3) |  |
| Diarrhea | 9 (7.8) | 2 (1.7) | 6 (7.5) | 1 (1.3) | 3 (8.3) | 1 (2.8) |
| Hypothyroidism | 8 (6.9) |  | 8 (10.0) |  |  |  |
| Anorexia | 7 (6.0) | 1 (0.9) | 7 (8.8) | 1 (1.3) |  |  |
| Lipase increase | 5 (4.3) | 3 (2.6) | 5 (6.3) | 3 (3.8) |  |  |
| Diabetes mellitus | 4 (3.4) | 1 (0.9) | 4 (5.0) | 1 (1.3) |  |  |
| Hyperthyroidism | 3 (2.6) | 1 (0.9) | 3 (3.8) | 1 (1.3) |  |  |
| Vomiting | 2 (1.7) |  | 2 (2.5) |  |  |  |

^a^ Nivolumab (1 mg/kg) plus Ipilimumab (3 mg/kg) every 3 weeks (four doses), followed by nivolumab (240 mg) monotherapy every 2 weeks.

^b^ Nivolumab (3 mg/kg) plus Ipilimumab (1 mg/kg) every 3 weeks (four doses), followed by nivolumab (240 mg) monotherapy every 2 weeks.

**Supplementary Table 4. Correlation between the occurrence of immune-related adverse events (irAEs) during prior atezolizumab plus bevacizumab therapy and subsequent nivolumab plus ipilimumab treatment.**

|  |  | **irAEs to Nivo/Ipi** | |  |
| --- | --- | --- | --- | --- |
|  |  | **No (n=35)** | **Yes (n=7)** |  |
| **irAEs to prior Ate/Bev** | **No (n=39)** | 32 (91.4) | 7 (100.0) |  |
|  | **Yes (n=3)** | 3 (8.6) | 0 | p = 1.00 |
